# Is It Worth Assessing the Prevalence of Sarcopenia in Pregnant Women? Should Any Impact on Pregnancy Outcomes Be Expected?

**DOI:** 10.3390/nu17162682

**Published:** 2025-08-19

**Authors:** Christian Göbl, Angela Dardano, Giuseppe Daniele, Andrea Tura

**Affiliations:** 1 Department of Obstetrics and Gynaecology, Division of Obstetrics and Feto-Maternal Medicine, Medical University of Vienna, 1090 Vienna, Austria; christian.goebl@meduniwien.ac.at; 2 Department of Clinical and Experimental Medicine, Section of Metabolic Diseases and Diabetes, University of Pisa, 56126 Pisa, Italy; angela.dardano@unipi.it (A.D.); giuseppe.daniele@unipi.it (G.D.); 3 CNR Institute of Neuroscience, 35127 Padova, Italy

**Keywords:** sarcopenia, pregnancy, gestational diabetes, obesity, perinatal outcomes, maternal complications, health care expenditure

## Abstract

The present article is an opinion piece mainly based on selected articles in the field of sarcopenia, with possible relevance for pregnancy. Sarcopenia has gained increasing interest in recent years, since it has emerged that sarcopenia may determine significant health consequences, with related substantial health care expenditure. In particular, some studies suggested that sarcopenia may cause increased risk for several diseases, such as type 2 diabetes, obesity, and major cardiovascular events. On the other hand, some studies have reported that the association between sarcopenia and these diseases may be bidirectional. In particular, this holds for type 2 diabetes, because sarcopenia and type 2 diabetes share many etiological and pathogenetic factors, such as insulin resistance, oxidative stress, low-grade chronic inflammation, and adiposity. It is also worth noting that some studies have shown a non-negligible sarcopenia prevalence even in people below 40 years of age, and therefore of reproductive age. Taken together, the above considerations support the hypothesis that sarcopenia may be present in women with gestational diabetes (GDM), which shares common traits with type 2 diabetes. Notably, we hypothesize that sarcopenia may exacerbate GDM-related complications and may influence maternal–fetal outcomes, such as preterm birth or cesarean delivery. Additionally, since pregnancy often presents with insulin resistance independently of any comorbidity, it is plausible that sarcopenia may be present during pregnancy even in cases of normal glycemia. However, there is a lack of data about sarcopenia prevalence in pregnancy and its potential impact on outcomes. Therefore, future studies addressing these aspects are advisable.

## 1. Sarcopenia: Several Potential Health Consequences with Remarkable Economic Burden

Sarcopenia is characterized by low skeletal muscle mass, strength, and performance, with a potential risk of several adverse health outcomes [1,2]. Sarcopenia has gained significant attention in recent years.

What are the reasons for the growing interest in sarcopenia? In our opinion, this is mainly due to the different severe health consequences that sarcopenia may cause, along with the significant health care expenditure related to it. As an example, a systematic review [3] concluded that most of the analyzed studies suggested significantly increased health care costs for people with sarcopenia compared to those without sarcopenia. It must be observed that few studies accounted for confounding factors that impact health care resource use irrespective of sarcopenia, such as age, sex, number of comorbidities, and nutritional status. Additionally, the periods considered for health expenditure assessment typically varied among studies. Nonetheless, as an example, some data reported in the review [3] indicated that the median hospital cost for patients undergoing surgery was approximately USD 39,000 for sarcopenic patients and USD 24,000 for non-sarcopenic patients. Notably, the difference remained statistically significant after adjusting for several patient and disease characteristics.

Why may sarcopenia cause the illustrated economic burden? As mentioned above, this is mainly due to the potential health consequences of sarcopenia that have emerged in recent years. It has to be acknowledged that the temporal nature of the relationship between sarcopenia and health consequences is not totally understood. Therefore, the relationship between sarcopenia and increased health care expenditure should be described as associative. In any case, a recent review study [4] reported a broad list of potential health consequences of sarcopenia, including functional, metabolic, and cognitive adverse outcomes. The review [4] highlighted that the focus on sarcopenia’s consequences was heterogeneous among studies and differed between studies in unhealthy subjects and in the general population. Despite these limitations, several potential consequences were indicated, such as risk for metabolic syndrome, diabetes (especially type 2, T2D), nonalcoholic fatty liver disease, hypertension, dysphagia, increased risk of major cardiovascular events, cognitive impairment, osteoporosis, falls, hospitalization, postoperative complications, and increased risk of mortality. It is notable that the causality in the association between sarcopenia and the reported health consequences remains uncertain, but the role of sarcopenia, at least as a contributing factor for the risk of such consequences, appears possible. In addition, a bidirectional relationship (crosstalk) has been described between sarcopenia and some of the aforementioned diseases, such as T2D (as discussed in some detail later), this meaning that sarcopenia may contribute to the risk of these diseases and vice versa. Additionally, a peculiar relationship exists between sarcopenia and obesity. Indeed, the association between sarcopenia and obesity has been defined specifically as sarcopenic obesity [5]. It has to be noted that patients with sarcopenic obesity may benefit from the so-called “obesity paradox”. Indeed, obesity is typically associated with unfavorable health consequences. However, in patients with sarcopenia, obesity may be, in contrast, a favorable condition, and this phenomenon has been indicated as the “obesity paradox”. In fact, it has been reported that elderly sarcopenic patients with different chronic diseases and a high body mass index show more favorable prognoses compared to subjects who have normal or low weight [6], summarized as delayed biological aging [7]. On the other hand, several health consequences of sarcopenic obesity have been reported, some of which are similar to those previously indicated for pure sarcopenia [8]. However, it is also interesting to note that sarcopenic obesity and pure sarcopenia differ slightly in terms of health consequences. Compared to pure sarcopenia, sarcopenic obesity showed a lower risk of fractures and a tendency toward a better nutritional status, but a higher degree of inflammation and a higher risk of dysglycemia [9]. Notably, a recent longitudinal study reported in sarcopenic obese patients a risk for progression from normoglycemic status to diabetes that was essentially doubled as compared to the risk in patients with sarcopenia or obesity alone [10].

In summary, the above considerations can be outlined as follows: (i) Sarcopenia is currently of major interest to health care systems due to the possible risk of health consequences and related health care expenditure; (ii) sarcopenia shares several factors with T2D, as well as with obesity. These factors explain the interest in investigating the prevalence of sarcopenia in gestational diabetes (GDM) and possibly in pregnancy in general. However, to fully support our hypothesis about the relationship between GDM and sarcopenia, one additional factor must be considered: the prevalence of sarcopenia in different age groups.

## 2. Can Sarcopenia Be Present in People of Reproductive Age?

At first glance, sarcopenia appears to primarily affect elderly people. However, sarcopenia can also be identified in people of non-elderly age, including those of reproductive age. A recent study assessed sarcopenia prevalence in participants aged 21–90 years [11]. Participants were stratified into several age groups (21–30, 31–40, 41–50, 51–60, 61–65, 66–70, 71–75, 76–80, and ≥81 years) and according to different criteria for sarcopenia definition. Specifically, when considering the recent (2019) criteria from the Asian Working Group for Sarcopenia [12], in the three age groups up to 50 years, the prevalence in males of probable sarcopenia (low muscle strength [2]) was found to be 10.7% (21–30 years), 23.1% (31–40 years), and 20% (41–50 years), respectively. In females, in the same age groups, the prevalence of probable sarcopenia was 6.3%, 11.4%, and 10.3%, respectively, and the prevalence of confirmed sarcopenia (low muscle strength and mass [2]) was also not negligible (9.4%, 11.4%, 2.6%). Notably, in the entire studied cohort, the prevalence of confirmed sarcopenia was 13.7% in males and 15.2% in females, with these values also suggesting gender-specific prevalence and risk factors.

## 3. The Reasons Why Sarcopenia Can Be Present During Pregnancy

As mentioned earlier, there is a crosstalk between sarcopenia and T2D, indicating that the presence of T2D increases the risk for sarcopenia and vice versa [13,14]. Additionally, a crosstalk has also been described between sarcopenia and obesity [15].

The crosstalk between sarcopenia and T2D arises from shared etiological and pathogenetic factors, such as insulin resistance, decreased levels of insulin-like growth factor 1 (IGF-1), mitochondrial dysfunction, oxidative stress, accumulation of advanced glycation end products (AGEs), low-grade chronic inflammation, and adiposity [16]. On the other hand, GDM is widely recognized as sharing etiological and pathogenetic factors with T2D [17,18]. Consequently, GDM potentially shares these factors with sarcopenia as well. Furthermore, insulin resistance, one of the most relevant factors shared by sarcopenia and T2D, is often exacerbated in GDM and may be present during pregnancy even in the absence of dysglycemia [19,20,21]. Additionally, hormonal changes during pregnancy may be protective but also harmful for sarcopenia risk, due to the increased levels of estrogens and cortisol. Specifically, increased estrogen levels are potentially protective, especially for the beneficial effects on mitochondrial metabolism [22,23,24], whereas increased cortisol levels are potentially harmful, since cortisol hypersecretion contributes to muscle breakdown [25,26,27,28]. Placental hormones may also affect muscle health by exacerbating insulin resistance, which can lead to muscle mass loss and atrophy [29,30]. Progesterone has been reported to impact the muscle condition, with this contributing to the pregnancy-associated changes in glucose homeostasis [31]. Other factors may also influence sarcopenia risk during pregnancy. Physical activity may decrease, especially in cases of obesity before pregnancy or abnormal weight gain during pregnancy. It may also decrease in association with pregnancy-related conditions, such as cervical insufficiency [32,33]. Inadequate nutrition or pregnancy eating disorders (like pregorexia, i.e., a fear of excessive weight gain during the pregnancy period [34,35]), and related alterations in gut microbiota [36,37], may also impact on sarcopenia risk [38,39]. Finally, increased protein demand for fetal growth, altered nutrient partitioning (leading to maternal protein deficiency), and inflammatory shifts could also alter muscle protein synthesis and breakdown [40,41,42]. Of note, since during fetal development there is a controlled distribution of nutrients, skeletal muscle, as well as adipose tissue, has a lower priority compared to the brain and heart. Therefore, skeletal muscle and adipose tissue development is particularly vulnerable to maternal nutritional deficiencies, which may be a consequence of altered nutrient partitioning [43,44].

On the other hand, it should be noted that, according to some studies, the prevalence of GDM may exceed 10% in some countries, and it is increasing worldwide [45,46,47,48,49]. In addition, as discussed above, we expect that sarcopenia may be present in pregnancy even when uncomplicated by dysglycemia. This potentially results in a large pool of women with sarcopenic pregnancies. In essence, during pregnancy, potentially even without GDM or obesity, since sarcopenia has a non-negligible prevalence in people of reproductive age, we state that the aforementioned etiological and pathogenetic factors may lead to sarcopenia or at least probable sarcopenia.

## 4. Why Does Sarcopenia Matter in Pregnancy?

Given the above premises, one should ask the following: if sarcopenia prevalence in pregnancy (possibly accompanied by GDM and/or obesity) is not negligible, is it worth recognizing? To answer this question, it is important to note that remarkable efforts are still being made to identify biomarkers of pregnancy complications and adverse fetal, neonatal, and perinatal outcomes [50,51]. Advanced methodologies for data analysis have recently been used to identify some GDM subtypes based on basic clinical information typically available in routine clinical practice, with different risks for complications and adverse outcomes [52]. Nevertheless, there is room for improvement in predicting the risk of pregnancy complications and adverse outcomes, with special interest in gestational hypertension, maternal delivery complications (preterm birth or cesarean delivery), and fetal complications (macrosomia, large or small for gestational age, and neonatal intensive care unit admission). To this end, one aspect that may be missing is the assessment of skeletal muscle quantity and quality during gestation, including muscle mass, muscle strength, and physical performance, these factors defining sarcopenia. One recent study showed some relationships between muscle strength (specifically, handgrip strength) and some pregnancy outcomes, such as, in fact, GDM onset or spontaneous abortion [53], but the investigators concluded that further research is needed. On the other hand, to our knowledge, no studies have assessed the impact of the main muscle-related factors (specifically, the sarcopenia factors) on pregnancy outcomes. Since these factors can be evaluated using methodologies that are inexpensive and safe even for pregnancy (bioimpedance analysis, handgrip and walk tests), future studies should address sarcopenia identification during pregnancy, especially when complicated by GDM or obesity, and assess related risks.

## 5. Are Interventions Possible in Cases of Sarcopenia in Pregnancy?

Targeted interventions have been shown to be effective for non-pregnant patients with sarcopenia or at high risk for it. These interventions include protein and/or micronutrient supplementation and specific physical exercise prescriptions [54,55,56,57]. Regarding physical exercise, some studies have specifically focused on the potential benefits for women, even of non-elderly age. For instance, a physical training program involving aerobic exercise using a cycle ergometer and walking has been shown to be remarkably effective for obese women with an average age of 53 years [58]. A similar physical training program appears feasible in pregnancy as well. Regarding nutritional interventions, some details are provided in the next section. Additionally, pharmacological intervention has recently been hypothesized, and the potential effects of antidiabetic medications have been reported, as outlined in a review study [59]. That study reported that several antidiabetic agents were investigated for their ability to prevent or treat sarcopenia due to their potential effects on muscle mass or, in general, on body composition (although not all agents are usable during pregnancy). These agents include insulin, insulin secretagogues, metformin, thiazolidinediones, GLP-1 receptor agonists, and DPP-IV, SGLT2, and alpha-glucosidase inhibitors [59]. This may also be important for the preconception care of women of reproductive age, possibly in the context of infertility treatments. Furthermore, if sarcopenia is identified in pregnancy, it may be interesting to assess whether gestational sarcopenia is an independent risk factor for later T2D onset.

## 6. Specific Information on Nutritional Interventions for Sarcopenia

We will now present some details on nutritional interventions for sarcopenia, with a focus on interventions for patients with sarcopenia associated with T2D. Then, we will consider the feasibility of such interventions in GDM and, more generally, in pregnancy.

In the recent study by Gaglio et al. [54], around two hundred patients with T2D were enrolled, screened for sarcopenia, and provided with personalized dietary plans. The plans aimed to achieve a daily energy intake of 25–30 kcal per kg of body weight and a daily protein intake of at least 1.1 g per kg of body weight. After six months of treatment, the group of T2D patients with a diagnosis of sarcopenia (16% of the total) showed an improvement in handgrip strength, in addition to a decrease in T2D severity, as reflected by glycated hemoglobin levels. Accordingly, another recent study [60], on over 1500 patients with T2D, showed that those patients without sarcopenia and obesity had a higher proportion of dietary supplement intake, higher nutritional education, and higher percentage of physical activity engagement, as compared to the patients with sarcopenia, with or without obesity. Another study on over 400 patients with T2D [61], 21% of whom had sarcopenia, found similar results: sarcopenic patients exhibited lower values in dietary assessment, walking time, and energy expenditure. Another study [62] examined a large cohort of nearly three thousand participants aged 65 years or older. Of those, 261 participants were diagnosed with T2D, and 126 participants also showed sarcopenia. Consistent with previous reports, total energy, protein, and carbohydrate intakes were lower in T2D patients with sarcopenia than in patients with T2D alone. Additionally, serum vitamin D levels were lower in patients with T2D and sarcopenia, suggesting the need for vitamin D supplementation. Similar findings concerning vitamin D were reported in another study in patients with T2D [63]. That study again found lower serum vitamin D levels in patients with sarcopenia, and highlighted the potential link between sarcopenia, low serum vitamin D levels, and low daily energy intake. Some review studies summarized the illustrated concepts. One review study [64] emphasized that elderly people require a daily calorie intake exceeding 30 kcal/kg to ensure muscle synthesis, combined with a protein intake of at least 1 g/kg (also through branched amino acid supplements), as well as vitamin D and polyunsaturated (omega-3) fatty acid supplementation. Similar concepts were reported in another review study [65], which, in addition, indicated that other vitamins may play a role in sarcopenia, as also reported elsewhere [57] (specifically, vitamins C, B6, B12, A, and E). Selenium, magnesium, calcium, potassium, phosphorus, iron, and zinc supplementation may also be useful against sarcopenia, as they may be associated with muscle performance and bone health [66,67].

## 7. Are Nutritional Interventions for Sarcopenia Feasible in Pregnancy?

One might wonder if the aforementioned nutritional strategies are feasible in GDM and in pregnancy in general. The answer is usually yes. Optimal daily energy intake levels, as reported in some review studies [68,69], essentially align with levels suggested for sarcopenia prevention, despite requiring possible adjustment among pregnancy trimesters. A similar conclusion also holds for protein intake [68], although some studies emphasized the importance of limiting animal protein intake during pregnancy, since animal protein consumption has been associated with GDM risk [70,71]. Regarding vitamin supplementation, a large body of studies exists, especially on vitamin D in both GDM and pregnancy with normal glycemic levels. Some review studies underscored the potential benefits of such supplementation, although it was generally recognized that the beneficial effects remain somewhat uncertain [72,73,74]. There is instead less evidence for the effects of omega-3 fatty acids, but one review study summarized the beneficial effects of omega-3 fatty acid (as well as vitamin) supplementation on the metabolic control of pregnant women with GDM [75]. Other review studies summarized the beneficial effects of mineral supplementation in pregnancy, particularly in GDM [76,77,78]. Therefore, we conclude that the main nutritional interventions appropriate for sarcopenia prevention or mitigation appear feasible, if not already proven as beneficial in pregnancy, especially in GDM. Thus, pregnant women with evidence of sarcopenia may benefit from nutritional interventions already known as appropriate for sarcopenia in other contexts (especially in patients with T2D).

## 8. Conclusions: The Need for Studies on Sarcopenia in Pregnancy

In conclusion, we have presented an opinion paper to stimulate interest in the possible presence and importance of sarcopenia during pregnancy. We have also discussed the relevance of sarcopenia for the health care system, due to the potentially associated health consequences and related care costs. Furthermore, we have explained why we suggest that sarcopenia may have a non-trivial prevalence in pregnancy even without GDM or obesity, and why sarcopenia may matter during pregnancy. However, it is important to note that, to our knowledge, there are no data on the prevalence of sarcopenia during pregnancy; therefore, our hypotheses remain speculative at present. In the coming months, we plan to assess the prevalence of sarcopenia (and probable sarcopenia) in pregnancy, as well as the possible related adverse pregnancy outcomes and postpartum consequences, based on an approved study protocol [79]. However, determining a specific definition for sarcopenia in pregnancy may be necessary, along with the validation of specific diagnostic criteria and the development of tailored screening strategies. Indeed, the pregnancy-related physiological changes may require setting different criteria cutoffs compared to those currently used for sarcopenia identification in non-pregnant women. In this context, interdisciplinary collaboration between professionals (such as obstetricians, endocrinologists, and nutritionists) should be encouraged. We also emphasize the need for longitudinal studies in the field, not limited to the pregnancy period but extended postpartum for sufficiently long periods, to assess long-term maternal health as well as offspring health. Finally, we point out that, in our opinion, other studies from different clinical centers and involving different ethnicities would be appropriate and timely.

## Data Availability

Not applicable.
